# (*Z*)-1-Di­phenyl­methyl-4-(3-phenyl­prop-2-en­yl)piperazine

**DOI:** 10.1107/S1600536814008289

**Published:** 2014-04-18

**Authors:** S. Shivaprakash, G. Chandrasekara Reddy, Jerry P. Jasinski

**Affiliations:** a Vittal Mallya Scientific Research Foundation, #94/3, 23rd Cross, 29th Main, BTM II Stage, Bangalore 560 076, India; b Department of Chemistry, Keene State College, 229 Main Street, Keene, NH 03435-2001, USA

## Abstract

In the title compound, C_26_H_28_N_2_, the piperazine group adopts a chair conformation with the exocyclic N—C bonds in equatorial orientations. The dihedral angle between the geminal benzene rings is 80.46 (12)° and the C=C—C—N torsion angle is 145.9 (2)°. In the crystal, weak C—H⋯π inter­actions link the mol­ecules into [100] chains.

## Related literature   

For the use of cinnerizine as an anti­histamine, see: Paton & Webster (1985 ▶). For synthetic methods of (*E*)-isomers of 1-benzhydryl-4-cinnamyl piperazines, see: Cignarella & Testa (1968 ▶). For the synthesis of the Z-isomer of cinnerizine, see: Shivaprakash & Chandrasekara Reddy (2014 ▶).
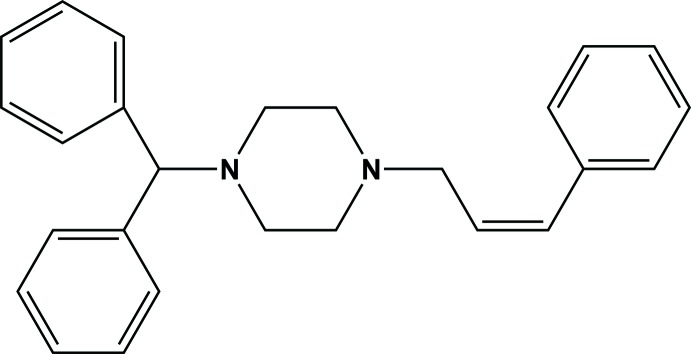


## Experimental   

### 

#### Crystal data  

C_26_H_28_N_2_
*M* *_r_* = 368.50
Monoclinic, 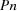

*a* = 8.7823 (3) Å
*b* = 9.6068 (3) Å
*c* = 12.4894 (4) Å
β = 94.834 (3)°
*V* = 1049.97 (6) Å^3^
*Z* = 2
Cu *K*α radiation
μ = 0.52 mm^−1^
*T* = 173 K
0.42 × 0.38 × 0.32 mm

#### Data collection  

Agilent Eos Gemini diffractometer
Absorption correction: multi-scan (*CrysAlis PRO* and *CrysAlis RED*; Agilent, 2012 ▶) *T*_min_ = 0.907, *T*_max_ = 1.000
6399 measured reflections
3316 independent reflections
3177 reflections with *I* > 2σ(*I*)
*R*_int_ = 0.027

#### Refinement  

*R*[*F* ^2^ > 2σ(*F* ^2^)] = 0.035
*wR*(*F* ^2^) = 0.096
*S* = 1.05
3316 reflections
254 parameters
2 restraints
H-atom parameters constrained
Δρ_max_ = 0.13 e Å^−3^
Δρ_min_ = −0.14 e Å^−3^
Absolute structure: Flack parameter determined using 1186 quotients [(*I* ^+^)−(*I* ^−^)]/[(*I* ^+^)+(*I* ^−^)] (Parsons *et al.*, 2013 ▶)
Absolute structure parameter: 0.1 (4)

### 

Data collection: *CrysAlis PRO* (Agilent, 2012 ▶); cell refinement: *CrysAlis PRO*; data reduction: *CrysAlis RED* (Agilent, 2012 ▶); program(s) used to solve structure: *SUPERFLIP* (Palatinus & Chapuis, 2007 ▶; Palatinus & van der Lee, 2008 ▶; Palatinus *et al.*, 2012 ▶); program(s) used to refine structure: *SHELXL2012* (Sheldrick, 2008 ▶); molecular graphics: *OLEX2* (Dolomanov *et al.*, 2009 ▶); software used to prepare material for publication: *OLEX2*.

## Supplementary Material

Crystal structure: contains datablock(s) I. DOI: 10.1107/S1600536814008289/hb7219sup1.cif

Structure factors: contains datablock(s) I. DOI: 10.1107/S1600536814008289/hb7219Isup2.hkl

Supporting information file. DOI: 10.1107/S1600536814008289/hb7219Isup3.cml

CCDC reference: 997025

Additional supporting information:  crystallographic information; 3D view; checkCIF report

## Figures and Tables

**Table 1:** Hydrogen-bond geometry (Å, °) *Cg*1 is the centroid of the C15–C20 ring.

*D*—H⋯*A*	*D*—H	H⋯*A*	*D*⋯*A*	*D*—H⋯*A*
C24—H24⋯*Cg*1^i^	0.95	2.70	3.629 (3)	164

Symmetry code: (i) 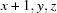.

**Fig. 1.**
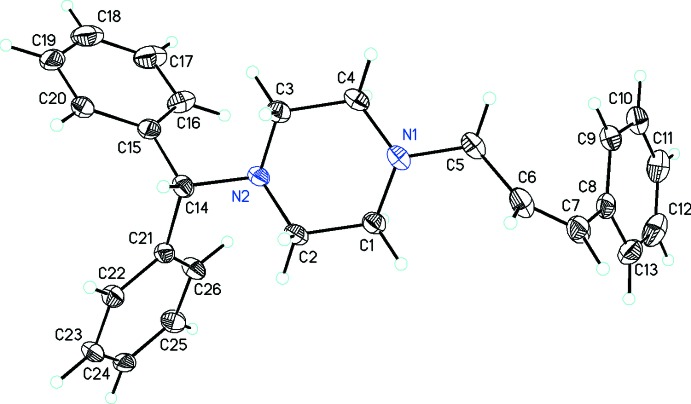
ORTEP drawing of (I), C26H28N2, showing 30% probability displacement ellipsoids.

## supplementary crystallographic information

### 1. Comment

Cinnerizine: (E)-1-(Diphenyl)methyl)-4-(3-phenyl-2-propenyl)piperazine is marketed as stugeron which is used as antihistamine (Paton & Webster, 1985). Because of greater biological importance of (E)-isomers of 1-benzhydryl-4-cinnamyl piperazines, several synthetic methods are described (Cignarella & Testa, 1968). But only recently the synthesis of (Z)-1-(Diphenylmethyl)-4-(3-phenyl-2-propenyl)piperazine is reported (Shivaprakash & Chandrasekara Reddy, 2014).

The title compound, C_26_H_28_N_2_, (I), is a close analogue of an existing drug viz., Cinnarizine, which has (E) geometry. We have prepared for the first time the (Z) isomer to study the structure activity relationship. However there is no report of any crystallographic data for this molecule so far. Hence this study was performed to confirm its structure. This compound exists as solid in free base form which could be crystallized easily. In continuation of our work in this area, we report here the crystal structure of (I).

In (I), the piperazine group adopts a slightly distorted chair conformation (puckering parameters Q, θ, and φ = 0.597 (2)Å, 3.95 (19)° and 168 (3)°, respectively (Fig. 1). The dihedral angles between the mean planes of the two methyl diphenyl groups (C15–C20 and C21–C26) with that of the 2-propenyl phenyl group (C8–C13) are 35.2 (1)° and 45.8 (8)°, respectively. The two methyl phenyl groups are separated by 80.4 (6)° with respect to each other.

### 2. Experimental

To a solution of 1-benzhydryl-4-(2-acetaldehyde) piperazine (5.0 g, 17.0 mmol) in dichloromethane (50 ml) under N_2_ atmosphere was added benzyltriphenyl phosphonium chloride (6.9 g, 17.9 mmol). The mixture was cooled to 278°K and t-BuOK (4.6 g, 41.3 mmol) was added under stirring. After completion, the reaction mass was quenched into water (100 ml). The organic layer was separated, dried over anhydrous sodium sulphate and concentrated under vacuum which was then subjected to column chromatography over silica gel with a EtOAc/Hexane mixture to afford the pure form of (Z)-1-benzhydrl-4-cinnamylpiperazine which was crystallized using absolute ethanol, white solid, mp: 363-365 K.

### 3. Refinement

All of the H atoms were placed in their calculated positions and then refined using the riding model with Atom—H lengths of 0.95Å (CH) or 0.99Å (CH_2_). Isotropic displacement parameters for these atoms were set to 1.2 (CH, CH_2_)times *U*_eq_ of the parent atom.

### Crystal data

**Table:**

C_26_H_28_N_2_	*F*(000) = 396
*M**_r_* = 368.50	*D*_x_ = 1.166 Mg m^−^^3^
Monoclinic, *P**n*	Cu *K*α radiation, λ = 1.54184 Å
*a* = 8.7823 (3) Å	Cell parameters from 3666 reflections
*b* = 9.6068 (3) Å	θ = 3.6–71.1°
*c* = 12.4894 (4) Å	µ = 0.52 mm^−^^1^
β = 94.834 (3)°	*T* = 173 K
*V* = 1049.97 (6) Å^3^	Irregular, colourless
*Z* = 2	0.42 × 0.38 × 0.32 mm

### Data collection

**Table:**

Agilent Eos Gemini diffractometer	3316 independent reflections
Radiation source: Enhance (Cu) X-ray Source	3177 reflections with *I* > 2σ(*I*)
Graphite monochromator	*R*_int_ = 0.027
Detector resolution: 16.0416 pixels mm^-1^	θ_max_ = 71.0°, θ_min_ = 4.6°
ω scans	*h* = −10→9
Absorption correction: multi-scan (*CrysAlis PRO* and *CrysAlis RED*; Agilent, 2012)	*k* = −11→9
*T*_min_ = 0.907, *T*_max_ = 1.000	*l* = −15→14
6399 measured reflections	

### Refinement

**Table:**

Refinement on *F*^2^	H-atom parameters constrained
Least-squares matrix: full	*w* = 1/[σ^2^(*F*_o_^2^) + (0.0592*P*)^2^ + 0.0586*P*] where *P* = (*F*_o_^2^ + 2*F*_c_^2^)/3
*R*[*F*^2^ > 2σ(*F*^2^)] = 0.035	(Δ/σ)_max_ < 0.001
*wR*(*F*^2^) = 0.096	Δρ_max_ = 0.13 e Å^−^^3^
*S* = 1.05	Δρ_min_ = −0.14 e Å^−^^3^
3316 reflections	Extinction correction: *SHELXL2012* (Sheldrick, 2008), Fc^*^=kFc[1+0.001xFc^2^λ^3^/sin(2θ)]^-1/4^
254 parameters	Extinction coefficient: 0.0072 (12)
2 restraints	Absolute structure: Flack parameter determined using 1186 quotients [(*I*^+^)-(*I*^-^)]/[(*I*^+^)+(*I*^-^)] (Parsons *et al.*, 2013)
Primary atom site location: structure-invariant direct methods	Absolute structure parameter: 0.1 (4)
Hydrogen site location: inferred from neighbouring sites	

### Special details

**Table:**

Experimental. ^1^H NMR: δ 7.10 - 7.42 (m, 15 H, Ar-H), 6.55 (d, J =12.0 Hz, 1 H), 5.77 (ddd, J =12.0, 6.6 Hz, 1 H), 4.22 (s, 1 H), 3.28 (dd, J = 6.6, 1.80 Hz, 2 H), 2.46 (bs, 8 H). ^13^C NMR: δ 142.8, 137.1, 131.6, 129.5, 128.9, 128.4, 128.1, 127.9, 126.9, 126.8, 76.2, 56.2, 53.5, 51.9. HRMS calculated for C_26_H_28_N_2_ [M+H] + 369.2331; found 369.2335.
Geometry. All esds (except the esd in the dihedral angle between two l.s. planes) are estimated using the full covariance matrix. The cell esds are taken into account individually in the estimation of esds in distances, angles and torsion angles; correlations between esds in cell parameters are only used when they are defined by crystal symmetry. An approximate (isotropic) treatment of cell esds is used for estimating esds involving l.s. planes.

### Fractional atomic coordinates and isotropic or equivalent isotropic displacement parameters (Å^2^)

**Table:**

	*x*	*y*	*z*	*U*_iso_*/*U*_eq_	
N1	0.5869 (2)	0.85143 (18)	0.65925 (14)	0.0334 (4)	
N2	0.72189 (19)	0.64457 (18)	0.80568 (14)	0.0305 (4)	
C1	0.7507 (2)	0.8187 (2)	0.66743 (17)	0.0328 (5)	
H1A	0.8089	0.9024	0.6492	0.039*	
H1B	0.7703	0.7447	0.6152	0.039*	
C2	0.8049 (2)	0.7707 (2)	0.77981 (17)	0.0340 (5)	
H2A	0.9159	0.7512	0.7841	0.041*	
H2B	0.7867	0.8448	0.8323	0.041*	
C3	0.5597 (3)	0.6811 (2)	0.80277 (18)	0.0359 (5)	
H3A	0.5452	0.7557	0.8556	0.043*	
H3B	0.5001	0.5988	0.8225	0.043*	
C4	0.5024 (2)	0.7302 (2)	0.69132 (17)	0.0344 (5)	
H4A	0.5132	0.6539	0.6392	0.041*	
H4B	0.3925	0.7539	0.6903	0.041*	
C5	0.5349 (3)	0.8889 (2)	0.54764 (18)	0.0377 (5)	
H5A	0.4226	0.9018	0.5414	0.045*	
H5B	0.5592	0.8123	0.4990	0.045*	
C6	0.6101 (3)	1.0202 (2)	0.5143 (2)	0.0442 (6)	
H6	0.6307	1.0881	0.5689	0.053*	
C7	0.6514 (3)	1.0529 (2)	0.4176 (2)	0.0459 (6)	
H7	0.7040	1.1386	0.4117	0.055*	
C8	0.6241 (3)	0.9702 (2)	0.31837 (19)	0.0385 (5)	
C9	0.4864 (3)	0.9022 (2)	0.29280 (18)	0.0377 (5)	
H9	0.4093	0.9051	0.3418	0.045*	
C10	0.4595 (3)	0.8300 (2)	0.19678 (19)	0.0448 (6)	
H10	0.3638	0.7852	0.1806	0.054*	
C11	0.5692 (4)	0.8225 (3)	0.1250 (2)	0.0551 (7)	
H11	0.5501	0.7723	0.0598	0.066*	
C12	0.7067 (4)	0.8881 (3)	0.1485 (2)	0.0614 (8)	
H12	0.7835	0.8824	0.0994	0.074*	
C13	0.7345 (3)	0.9627 (3)	0.2430 (2)	0.0522 (7)	
H13	0.8294	1.0095	0.2572	0.063*	
C14	0.7777 (2)	0.5848 (2)	0.91021 (16)	0.0321 (5)	
H14	0.7564	0.6523	0.9681	0.039*	
C15	0.6962 (2)	0.4481 (2)	0.93040 (19)	0.0370 (5)	
C16	0.6542 (3)	0.3558 (3)	0.8466 (2)	0.0465 (6)	
H16	0.6703	0.3810	0.7749	0.056*	
C17	0.5894 (3)	0.2279 (3)	0.8667 (3)	0.0617 (8)	
H17	0.5625	0.1659	0.8089	0.074*	
C18	0.5639 (3)	0.1902 (3)	0.9704 (3)	0.0702 (10)	
H18	0.5183	0.1031	0.9842	0.084*	
C19	0.6051 (3)	0.2801 (4)	1.0533 (3)	0.0696 (10)	
H19	0.5892	0.2539	1.1249	0.083*	
C20	0.6700 (3)	0.4093 (3)	1.0340 (2)	0.0496 (6)	
H20	0.6962	0.4708	1.0921	0.060*	
C21	0.9488 (2)	0.5561 (2)	0.91718 (17)	0.0304 (4)	
C22	1.0405 (3)	0.5915 (2)	1.00883 (18)	0.0364 (5)	
H22	0.9979	0.6412	1.0649	0.044*	
C23	1.1937 (3)	0.5551 (3)	1.0193 (2)	0.0433 (6)	
H23	1.2553	0.5794	1.0828	0.052*	
C24	1.2572 (3)	0.4838 (2)	0.9382 (2)	0.0416 (6)	
H24	1.3622	0.4585	0.9457	0.050*	
C25	1.1672 (3)	0.4492 (3)	0.8459 (2)	0.0433 (5)	
H25	1.2102	0.4001	0.7898	0.052*	
C26	1.0146 (3)	0.4860 (2)	0.83531 (19)	0.0389 (5)	
H26	0.9538	0.4631	0.7712	0.047*	

### Atomic displacement parameters (Å^2^)

**Table:**

	*U*^11^	*U*^22^	*U*^33^	*U*^12^	*U*^13^	*U*^23^
N1	0.0309 (10)	0.0336 (8)	0.0341 (9)	0.0054 (7)	−0.0063 (7)	−0.0034 (7)
N2	0.0229 (9)	0.0342 (8)	0.0337 (9)	0.0008 (7)	−0.0025 (7)	0.0018 (7)
C1	0.0289 (11)	0.0311 (10)	0.0377 (11)	0.0010 (8)	−0.0015 (9)	0.0001 (8)
C2	0.0263 (11)	0.0351 (10)	0.0392 (11)	−0.0010 (8)	−0.0061 (8)	−0.0001 (9)
C3	0.0265 (11)	0.0424 (11)	0.0388 (11)	0.0038 (8)	0.0015 (9)	0.0017 (9)
C4	0.0242 (10)	0.0393 (10)	0.0386 (12)	0.0050 (8)	−0.0039 (8)	−0.0047 (9)
C5	0.0372 (13)	0.0371 (11)	0.0367 (12)	0.0065 (9)	−0.0089 (9)	−0.0044 (8)
C6	0.0520 (16)	0.0312 (10)	0.0456 (13)	0.0043 (10)	−0.0184 (11)	−0.0026 (9)
C7	0.0510 (16)	0.0298 (10)	0.0540 (15)	−0.0028 (10)	−0.0134 (12)	0.0067 (9)
C8	0.0442 (14)	0.0277 (9)	0.0427 (12)	0.0041 (9)	−0.0026 (10)	0.0105 (8)
C9	0.0391 (13)	0.0358 (11)	0.0376 (11)	0.0072 (9)	−0.0007 (9)	0.0030 (9)
C10	0.0541 (16)	0.0383 (11)	0.0403 (12)	0.0057 (10)	−0.0061 (11)	0.0019 (9)
C11	0.081 (2)	0.0448 (13)	0.0399 (14)	0.0096 (13)	0.0088 (13)	0.0032 (10)
C12	0.081 (2)	0.0510 (14)	0.0570 (16)	0.0110 (15)	0.0313 (16)	0.0143 (13)
C13	0.0481 (16)	0.0423 (12)	0.0671 (17)	−0.0029 (11)	0.0097 (12)	0.0179 (12)
C14	0.0285 (11)	0.0376 (10)	0.0298 (10)	0.0043 (9)	−0.0007 (8)	−0.0001 (8)
C15	0.0217 (10)	0.0467 (12)	0.0428 (12)	0.0061 (8)	0.0044 (9)	0.0095 (9)
C16	0.0382 (14)	0.0439 (12)	0.0590 (15)	−0.0051 (10)	0.0139 (11)	0.0017 (11)
C17	0.0424 (16)	0.0480 (14)	0.096 (2)	−0.0056 (12)	0.0120 (15)	0.0047 (14)
C18	0.0332 (14)	0.0609 (17)	0.116 (3)	−0.0057 (13)	0.0018 (16)	0.042 (2)
C19	0.0252 (13)	0.106 (3)	0.077 (2)	0.0017 (14)	0.0016 (12)	0.057 (2)
C20	0.0233 (12)	0.0780 (17)	0.0469 (14)	0.0040 (11)	−0.0009 (10)	0.0215 (13)
C21	0.0273 (11)	0.0285 (9)	0.0345 (10)	−0.0011 (7)	−0.0016 (8)	0.0052 (8)
C22	0.0364 (12)	0.0367 (10)	0.0349 (11)	−0.0057 (9)	−0.0034 (9)	0.0027 (9)
C23	0.0337 (12)	0.0505 (12)	0.0429 (13)	−0.0116 (10)	−0.0136 (10)	0.0092 (10)
C24	0.0233 (11)	0.0470 (12)	0.0533 (14)	−0.0029 (9)	−0.0032 (10)	0.0184 (10)
C25	0.0317 (12)	0.0492 (13)	0.0493 (13)	0.0060 (9)	0.0043 (10)	0.0017 (10)
C26	0.0287 (12)	0.0482 (12)	0.0385 (11)	0.0041 (9)	−0.0047 (9)	−0.0049 (9)

### Geometric parameters (Å, º)

**Table:**

N1—C1	1.467 (3)	C11—C12	1.372 (5)
N1—C4	1.456 (3)	C12—H12	0.9500
N1—C5	1.475 (3)	C12—C13	1.385 (4)
N2—C2	1.464 (3)	C13—H13	0.9500
N2—C3	1.465 (3)	C14—H14	1.0000
N2—C14	1.472 (3)	C14—C15	1.527 (3)
C1—H1A	0.9900	C14—C21	1.523 (3)
C1—H1B	0.9900	C15—C16	1.397 (4)
C1—C2	1.516 (3)	C15—C20	1.384 (3)
C2—H2A	0.9900	C16—H16	0.9500
C2—H2B	0.9900	C16—C17	1.386 (4)
C3—H3A	0.9900	C17—H17	0.9500
C3—H3B	0.9900	C17—C18	1.381 (5)
C3—C4	1.515 (3)	C18—H18	0.9500
C4—H4A	0.9900	C18—C19	1.373 (6)
C4—H4B	0.9900	C19—H19	0.9500
C5—H5A	0.9900	C19—C20	1.395 (5)
C5—H5B	0.9900	C20—H20	0.9500
C5—C6	1.499 (3)	C21—C22	1.385 (3)
C6—H6	0.9500	C21—C26	1.390 (3)
C6—C7	1.328 (4)	C22—H22	0.9500
C7—H7	0.9500	C22—C23	1.386 (3)
C7—C8	1.475 (3)	C23—H23	0.9500
C8—C9	1.388 (3)	C23—C24	1.380 (4)
C8—C13	1.409 (4)	C24—H24	0.9500
C9—H9	0.9500	C24—C25	1.382 (4)
C9—C10	1.388 (3)	C25—H25	0.9500
C10—H10	0.9500	C25—C26	1.381 (3)
C10—C11	1.372 (4)	C26—H26	0.9500
C11—H11	0.9500		
			
C1—N1—C5	110.01 (17)	C10—C11—H11	120.3
C4—N1—C1	109.22 (16)	C10—C11—C12	119.4 (3)
C4—N1—C5	109.34 (17)	C12—C11—H11	120.3
C2—N2—C3	107.32 (16)	C11—C12—H12	119.7
C2—N2—C14	112.58 (16)	C11—C12—C13	120.6 (3)
C3—N2—C14	111.44 (17)	C13—C12—H12	119.7
N1—C1—H1A	109.4	C8—C13—H13	119.5
N1—C1—H1B	109.4	C12—C13—C8	120.9 (3)
N1—C1—C2	111.06 (18)	C12—C13—H13	119.5
H1A—C1—H1B	108.0	N2—C14—H14	108.6
C2—C1—H1A	109.4	N2—C14—C15	110.89 (17)
C2—C1—H1B	109.4	N2—C14—C21	111.98 (17)
N2—C2—C1	109.45 (17)	C15—C14—H14	108.6
N2—C2—H2A	109.8	C21—C14—H14	108.6
N2—C2—H2B	109.8	C21—C14—C15	107.97 (17)
C1—C2—H2A	109.8	C16—C15—C14	121.3 (2)
C1—C2—H2B	109.8	C20—C15—C14	120.2 (2)
H2A—C2—H2B	108.2	C20—C15—C16	118.3 (2)
N2—C3—H3A	109.6	C15—C16—H16	119.5
N2—C3—H3B	109.6	C17—C16—C15	120.9 (3)
N2—C3—C4	110.14 (18)	C17—C16—H16	119.5
H3A—C3—H3B	108.1	C16—C17—H17	119.9
C4—C3—H3A	109.6	C18—C17—C16	120.2 (3)
C4—C3—H3B	109.6	C18—C17—H17	119.9
N1—C4—C3	111.38 (18)	C17—C18—H18	120.3
N1—C4—H4A	109.4	C19—C18—C17	119.3 (3)
N1—C4—H4B	109.4	C19—C18—H18	120.3
C3—C4—H4A	109.4	C18—C19—H19	119.5
C3—C4—H4B	109.4	C18—C19—C20	120.9 (3)
H4A—C4—H4B	108.0	C20—C19—H19	119.5
N1—C5—H5A	109.4	C15—C20—C19	120.3 (3)
N1—C5—H5B	109.4	C15—C20—H20	119.9
N1—C5—C6	111.06 (18)	C19—C20—H20	119.9
H5A—C5—H5B	108.0	C22—C21—C14	120.31 (19)
C6—C5—H5A	109.4	C22—C21—C26	118.6 (2)
C6—C5—H5B	109.4	C26—C21—C14	121.00 (19)
C5—C6—H6	116.1	C21—C22—H22	119.7
C7—C6—C5	127.8 (2)	C21—C22—C23	120.6 (2)
C7—C6—H6	116.1	C23—C22—H22	119.7
C6—C7—H7	116.6	C22—C23—H23	119.8
C6—C7—C8	126.8 (2)	C24—C23—C22	120.3 (2)
C8—C7—H7	116.6	C24—C23—H23	119.8
C9—C8—C7	121.6 (2)	C23—C24—H24	120.2
C9—C8—C13	117.2 (2)	C23—C24—C25	119.6 (2)
C13—C8—C7	121.1 (2)	C25—C24—H24	120.2
C8—C9—H9	119.5	C24—C25—H25	120.0
C10—C9—C8	121.0 (2)	C26—C25—C24	120.1 (2)
C10—C9—H9	119.5	C26—C25—H25	120.0
C9—C10—H10	119.6	C21—C26—H26	119.6
C11—C10—C9	120.8 (3)	C25—C26—C21	120.9 (2)
C11—C10—H10	119.6	C25—C26—H26	119.6
			
N1—C1—C2—N2	60.7 (2)	C9—C10—C11—C12	−0.5 (4)
N1—C5—C6—C7	145.9 (2)	C10—C11—C12—C13	−0.7 (4)
N2—C3—C4—N1	−59.4 (2)	C11—C12—C13—C8	1.5 (4)
N2—C14—C15—C16	35.6 (3)	C13—C8—C9—C10	0.0 (3)
N2—C14—C15—C20	−148.1 (2)	C14—N2—C2—C1	175.11 (17)
N2—C14—C21—C22	135.90 (19)	C14—N2—C3—C4	−174.99 (17)
N2—C14—C21—C26	−48.3 (3)	C14—C15—C16—C17	175.7 (2)
C1—N1—C4—C3	55.5 (2)	C14—C15—C20—C19	−175.6 (2)
C1—N1—C5—C6	−64.6 (2)	C14—C21—C22—C23	174.5 (2)
C2—N2—C3—C4	61.3 (2)	C14—C21—C26—C25	−174.2 (2)
C2—N2—C14—C15	−175.90 (17)	C15—C14—C21—C22	−101.7 (2)
C2—N2—C14—C21	−55.2 (2)	C15—C14—C21—C26	74.0 (3)
C3—N2—C2—C1	−61.9 (2)	C15—C16—C17—C18	0.7 (4)
C3—N2—C14—C15	63.4 (2)	C16—C15—C20—C19	0.9 (3)
C3—N2—C14—C21	−175.87 (17)	C16—C17—C18—C19	−0.8 (5)
C4—N1—C1—C2	−56.3 (2)	C17—C18—C19—C20	1.0 (4)
C4—N1—C5—C6	175.45 (19)	C18—C19—C20—C15	−1.1 (4)
C5—N1—C1—C2	−176.29 (16)	C20—C15—C16—C17	−0.7 (4)
C5—N1—C4—C3	175.89 (17)	C21—C14—C15—C16	−87.5 (2)
C5—C6—C7—C8	4.0 (4)	C21—C14—C15—C20	88.9 (2)
C6—C7—C8—C9	40.9 (4)	C21—C22—C23—C24	0.4 (3)
C6—C7—C8—C13	−142.3 (3)	C22—C21—C26—C25	1.6 (3)
C7—C8—C9—C10	177.0 (2)	C22—C23—C24—C25	0.3 (3)
C7—C8—C13—C12	−178.2 (2)	C23—C24—C25—C26	0.0 (3)
C8—C9—C10—C11	0.8 (3)	C24—C25—C26—C21	−0.9 (4)
C9—C8—C13—C12	−1.2 (3)	C26—C21—C22—C23	−1.4 (3)

### Hydrogen-bond geometry (Å, º)

Cg1 is the centroid of the C15–C20 ring.

**Table:**

*D*—H···*A*	*D*—H	H···*A*	*D*···*A*	*D*—H···*A*
C24—H24···*Cg*1^i^	0.95	2.70	3.629 (3)	164

Symmetry code: (i) *x*+1, *y*, *z*.
